# Interaction between mitochondrial oxidative stress and myocardial fibrosis in the context of diabetes

**DOI:** 10.3389/fendo.2025.1596436

**Published:** 2025-06-05

**Authors:** Pu-Hua Zhang, Nuo-Nan Li, Xiang Gu, Chun-Xia Zhou, Zhen-Zhen Jiang, Xian-Jun Luo, Hong-Wen Zhu, Xiao-Yong Zhu

**Affiliations:** ^1^ Department of Cardiology, Jiujiang University Affiliated Hospital, Jiujiang, Jiangxi, China; ^2^ School of Clinical Medicine, Gannan Medical University, Ganzhou, Jiangxi, China; ^3^ Jiujiang Clinical Precision Medicine Research Center, Jiujiang, Jiangxi, China

**Keywords:** diabetes, mitochondrial oxidative stress, myocardial fibrosis, signaling pathways, interaction, drug therapy

## Abstract

Diabetes represents a global chronic health issue and has emerged as a crucial risk factor for cardiovascular diseases (CVD). Myocardial fibrosis (MF), which often accompanies diabetes, plays a pivotal role in the progression of cardiac dysfunction and heart failure (HF). Recent research has highlighted mitochondrial oxidative stress (OS) as a fundamental mechanism driving MF in diabetic conditions. Elevated blood glucose levels and metabolic imbalances lead to mitochondrial impairments, which in turn cause an excessive buildup of reactive oxygen species (ROS), culminating in OS. This OS not only inflicts direct damage on myocardial cells but also facilitates the proliferation of myocardial fibroblasts and collagen accumulation through the activation of specific signaling pathways, thus intensifying MF. Furthermore, MF itself intensifies mitochondrial OS, creating a vicious cycle that ultimately impairs myocardial structure and function. Thus, a thorough understanding of the interaction between mitochondrial OS and MF in diabetes is crucial for identifying effective therapeutic targets and enhancing the early diagnosis and intervention strategies for diabetic cardiomyopathy.

## Graphical Abstract

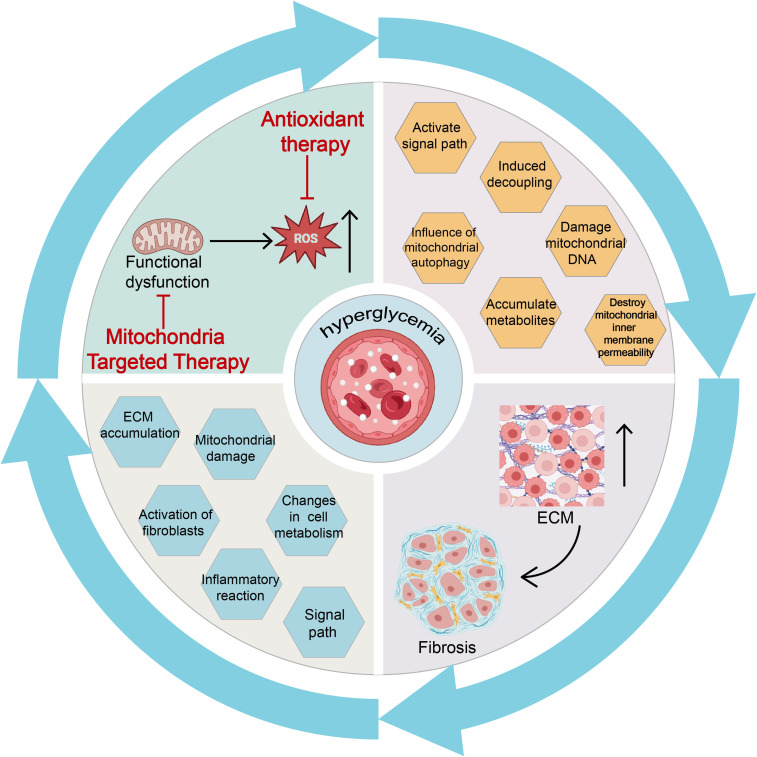

## 1 Introduction

Currently, approximately 540 million individuals worldwide suffer from diabetes, with around 90% diagnosed with type 2 diabetes (T2DM) (1). By 2045, this number is projected to rise to 629 million, significantly increasing the global public health burden (2). The diabetes pandemic places a substantial burden on society, not only due to high healthcare costs but also due to the deterioration of patients’ physical health (3). Cardiovascular disease (CVD) is the primary cause of morbidity and mortality among individuals with T2DM, with HF being the most prevalent manifestation (4). A comprehensive study involving 1.5 million participants revealed that, compared to those without diabetes, individuals with T2DM have a significantly higher risk (approximately 2.5-fold greater) of developing HF, thus exacerbating the negative impact of diabetes on cardiac function (5). T2DM is identified as a significant and independent predictor of new-onset HF within the general population (6). Diabetes cardiomyopathy (DCM), a distinct form of cardiomyopathy that excludes myocardial damage due to hypertension or coronary artery disease, is marked by abnormal myocardial cell metabolism and a progressive decline in cardiac functionality (7). Initially manifested as myocardial diastolic dysfunction, it progresses to systolic dysfunction in advanced stages, becoming a leading cause of HF in diabetic patients (8).

Mitochondria are crucial centers for energy metabolism and signaling regulation within cells, primarily responsible for producing adenosine triphosphate (ATP) through oxidative phosphorylation (OXPHOS), and playing vital roles in calcium homeostasis, fatty acid (FA) metabolism, and various cellular signaling pathways (9). Under typical physiological conditions, ROS production during mitochondrial metabolism is unavoidable, with cells depending on antioxidant systems to preserve a dynamic equilibrium of ROS levels. However, in diabetes, hyperglycemia and metabolic abnormalities compromise mitochondrial function and ATP synthesis, leading to excessive ROS production. This accumulation of ROS triggers OS, damages myocardial cells, activates inflammatory responses, and accelerates MF, thereby exacerbating cardiac dysfunction (10).

This review explores the interaction between mitochondrial OS and MF in the context of diabetes, covering mitochondrial function and damage mechanisms, the interplay between OS and fibrosis, and therapeutic strategies, thereby providing new insights for future treatments.

## 2 The basic function of mitochondria in cardiomyocytes and the background of MF in diabetes

### 2.1 The basic functions of mitochondria

Mitochondria are crucial organelles in cardiac myocytes, performing multiple essential functions, especially in energy supply and cellular homeostasis. The primary functions of mitochondria in myocardial cells are outlined below:

#### 2.1.1 ATP synthesis and energy supply

The heart’s continuous beating depends on a substantial supply of high-energy phosphate compounds, with ATP serving as the main energy source. The myocardium’s ATP consumption constitutes approximately 8% of the total ATP consumed by the human body, with around 95% produced by mitochondria through OXPHOS (11). Mitochondria comprise 30% to 40% of the volume of myocardial cells, reflecting the heart’s requirement for efficient energy metabolism (2, 12).

#### 2.1.2 FA oxidation

Under normal conditions, FA oxidation provides approximately 70% of cardiac energy, with the remainder derived from the oxidation of other nutrients such as glucose, ketones, lactate, and amino acids (13–15).It is important to note that FA oxidation consumes approximately 12% more oxygen than glucose oxidation to generate an equivalent amount of ATP (14).

#### 2.1.3 Calcium homeostasis regulation

Mitochondria are essential for maintaining Ca^2+^ homeostasis in myocardial cells. The dynamic equilibrium of Ca^2+^ between the sarcoplasmic reticulum (SR) and mitochondria significantly affects myocardial excitation-contraction coupling (ECC) (14, 16). During myocardial depolarization, Ca^2+^ first moves into the cytoplasm via L-type voltage-dependent calcium channels, subsequently triggering additional Ca^2+^ release from the SR, causing myocardial contraction. In the relaxation phase, excess Ca^2+^ is primarily transported back into the SR via SR Ca^2+^-ATPase, while some Ca^2+^ is expelled from cells through Na^+^/Ca^2+^ exchangers (14, 17).

#### 2.1.4 Free radical generation and clearance

OS is typified by a relative imbalance between excessive ROS production and inadequate antioxidant defense within the organism. Under standard physiological conditions, mitochondria generate ATP through OXPHOS, where the electron reduction process in the respiratory chain turns a minor portion of oxygen into ROS (18). Cells typically depend on the antioxidant system to maintain a dynamic balance of ROS and prevent oxidative damage (19).

### 2.2 Effects of diabetes on myocardial metabolism

In diabetic patients, increased serum FAs and triglycerides are commonly observed, promoting the uptake and oxidation of FAs. Research in various T2DM animal models (e.g., db/db mice, ob/ob mice, and Zucker diabetic fatty rats) has shown increased FA oxidation (FAO) rates and decreased glucose oxidation (20). Similar metabolic shifts have been observed in human diabetic patients, where FA uptake and oxidation are increased under conditions of insulin resistance (IR) or diabetic conditions, while insulin-mediated glucose uptake and utilization are decreased (20).

The elevation in FAO rates is partly due to increased activity of peroxisome proliferator-activated receptors (PPARs), particularly PPARα. In diabetes or IR, elevated myocardial FA levels activate PPAR α, which induces pyruvate dehydrogenase kinase (PDK) activity, thereby reducing glucose oxidation capacity and increasing mitochondrial FA uptake (21). However, since FA oxidation consumes approximately 12% more oxygen than glucose oxidation, this process can exacerbate hypoxia in myocardial microvascular disease.

Furthermore, lipid accumulation not only impacts myocardial energy metabolism but also leads to myocardial lipid toxicity. Metabolic intermediates arising from FA oxidation, such as ceramide, diacylglycerol (DAG), and acylcarnitine, play critical roles in the progression of diabetic cardiomyopathy (DCM) (22). Ceramide, in particular, acts as a detrimental intermediate that impairs cardiomyocyte function through several mechanisms. First, ceramide induces mitochondrial dysfunction; specifically, C2-ceramide disrupts mitochondrial activity by inhibiting Complex I of the electron transport chain (ETC), leading to elevated reactive oxygen species (ROS) generation and apoptosis (23). Additionally, ceramide is implicated in autophagic processes, notably mitophagy, by directing autophagosomes towards mitochondria, which inhibits ATP synthesis and triggers detrimental mitophagy (24), thereby causing cardiotoxicity through lipid accumulation.

Intermediate metabolites of FA metabolism, such as acyl CoA and acylcarnitine, can affect the mitochondrial ATP/ADP ratio and diminish mitochondrial metabolic function. High blood glucose levels exacerbate lipid and protein oxidation through mitochondrial OS, accelerating the accumulation of advanced glycation end products (AGEs) (25). AGEs not only directly alter myocardial structure but also disrupt the insulin signaling pathway, reduce nitric oxide (NO) synthesis, and increase MF. AGEs bind to their receptor (RAGE) and activate the mitogen-activated protein kinase (MAPK) pathway, enhancing the inflammatory response, promoting matrix protein and connective tissue deposition, and leading to myocardial injury (MI) (25).

### 2.3 Pathology of MF in diabetes

MF in diabetes represents a form of cardiac structural remodeling, with fibrosis as a principal pathological characteristic that directly impacts heart structure and function. Interstitial and perivascular fibrosis, notable early histological features, lead to decreased cardiac compliance. Following focal myocardial cell necrosis, a pro-inflammatory response from connective tissue cells induces fibrosis, impairing cardiac function. Diastolic dysfunction in diabetic patients is closely associated with MF (26). Metabolic disorders and microcirculation abnormalities can cause hypertrophy, atrophy, and even necrosis of myocardial cells, commonly observed in heart biopsies of diabetic patients (26). Long-term diabetes diminishes the number of myocardial fibers and ATPase activity, affecting myocardial contractility, reducing cardiac output, and increasing HF risk (27, 28). Collagen accumulation in the ventricle’s inner, middle, and outer layers leads to myocardial sclerosis, resulting in cardiac dysfunction and HF (29). In the initial stages of diabetes, cellular damage and diastolic dysfunction gradually progress to left ventricular hypertrophy and systolic dysfunction (30). Diabetes-induced microvascular remodeling is characterized by basement membrane thickening, hyaline arteriosclerosis, and capillary aneurysms, causing myocardial cell damage and interstitial fibrosis (31).

important to highlight that the cardiac impact of diabetes extends beyond mature cardiomyocytes and endothelial cells. Recent evidence suggests diabetic hearts exhibit features of cellular senescence, impaired cellular homeostasis, and inadequate replacement of apoptotic cells. These phenomena are intimately linked to sustained inflammation, oxidative stress (OS), and metabolic derangements, collectively resulting in persistent injury to cardiac stem cells and compromised myocardial regeneration and repair. Reduced quantity and impaired function of cardiac stem cells exacerbate deficits in myocardial tissue recovery, emerging as critical driving factors of diabetic myocardial fibrosis (MF). Therefore, targeting the dysregulated stem cell niche to facilitate stem cell activation or recovery may offer novel therapeutic strategies for diabetic cardiomyopathy (32).

## 3 Formation of mitochondrial OS and its role in diabetes

### 3.1 Basic concepts of OS

OS is defined as a state of redox imbalance due to excessive production of ROS or decreased antioxidant capacity. Superoxide anions (O_2_^-^), hydrogen peroxide (H_2_O_2_), hydroxyl radicals (OH·), and peroxynitrite (ONOO^-^), which result from the reaction between superoxide and NO, are collectively known as ROS (19). When ROS production exceeds effective clearance capabilities, OS occurs, potentially causing cell damage and functional abnormalities (19).

In the heart, mitochondria are the principal producers of ROS, which are generated via multiple pathways (33). Predominantly, ROS are produced by ETC-dependent and ETC-independent mechanisms. Within the ETC, the main sources include nonspecific electron leakage and the OXPHOS process at complexes I and III (34). Specifically, complex I produces O_2_^-^ at the flavin mononucleotide (FMN) site, and complex III generates ROS during its interaction with ubiquinone at the coQ site (20). Additionally, smaller quantities of ROS originate from other complexes or through reverse electron transfer (RET). Non-ETC sources such as monoamine oxidase (MAO) (35), which produces H_2_O_2_ during neurotransmitter deamination (20), and NADPH oxidase 4 (NOX4), which catalyzes the direct production of O_2_^-^, contribute to the increased oxidative burden. Accumulation of these ROS can precipitate OS and impair myocardial functionality.

Under normal conditions, mitochondria maintain ROS homeostasis through an antioxidant system, primarily involving antioxidant enzymes and molecular neutralizers of ROS. Manganese superoxide dismutase (MnSOD) converts O_2_^-^ to H_2_O_2_, subsequently reduced to water through catalase, the glutathione system (Gpx), peroxidase (Prx), and thioredoxin (Trx). This process is regulated by mitochondrial redox status and reducing equivalents. However, several studies have reported that during DCM, mitochondrial antioxidant capacity is significantly reduced, leading to redox imbalance and mitochondrial-induced OS, which further exacerbates myocardial damage (19, 36, 37).

### 3.2 Mechanisms of mitochondrial OS in diabetes

#### 3.2.1 Hyperglycemia

**Figure 1:**
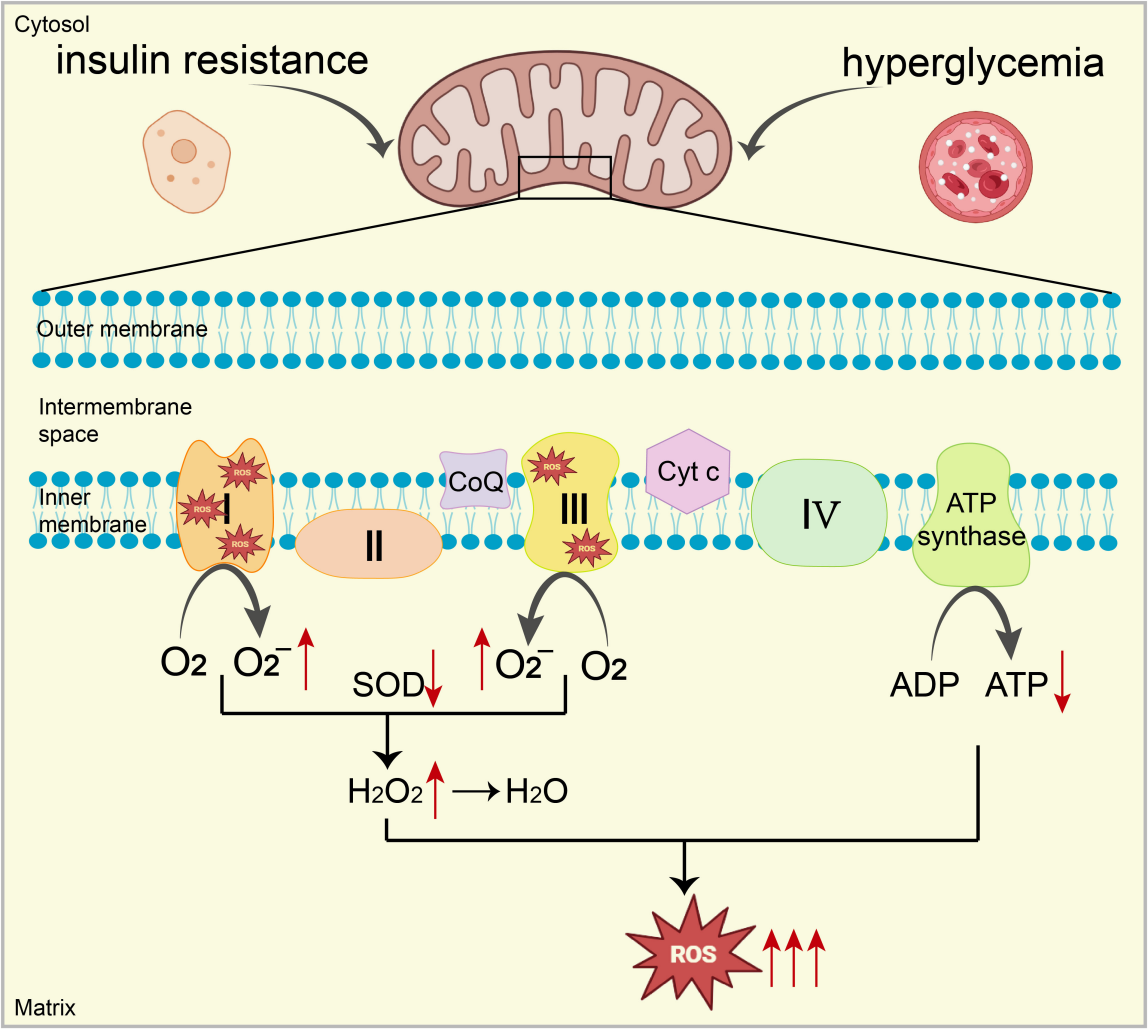
Under conditions of high blood sugar and IR, excessive electron leakage from ETC complexes I and III reacts with oxygen, increasing O_2_^-^ production. Additionally, mitochondrial antioxidant capacity (e.g., SOD) is significantly impaired, ultimately leading to a substantial increase in ROS, resulting in mitochondrial dysfunction. Mitochondrial dysfunction inhibits the expression and activity of key enzymes (complexes I, III, and IV), reducing ATP production. This decrease further increases ROS generation, creating a vicious cycle leading to mitochondrial OS.

The phenomenon of glucose toxicity from hyperglycemia is intimately linked with various cardiac conditions in diabetes, including AGE formation, fibrosis, aberrant inflammatory signaling, O-GlcNAc modification, and impaired Ca^2+^ processing. Hyperglycemia can trigger excessive ROS production in the diabetic heart via several mechanisms, including the activation of enzymes such as NOX, XO, and NOS (38–40). Elevated glucose levels also intensify the production of O_2_^-^, NO, ONOO⁻, and AGEs by boosting flux through the polyol and hexosamine pathways and by activating protein kinases (19). This rampant production of mitochondria-derived ROS, exacerbated by these pathways, promotes OS and contributes to the progression of cardiac disease in diabetes, highlighting the critical pathogenic role of ROS accumulation induced by hyperglycemia (19). (**Figure 1**)

#### 3.2.2 IR

Myocardial IR is a key factor in the structural and functional alterations of the heart, associated with systemic metabolic disorders such as hyperinsulinemia, hyperglycemia, and hyperlipidemia, and anomalies in myocardial insulin signaling (41). Initial changes in myocardial glucose uptake, marked by reduced GLUT4 expression and impaired translocation, often precede dysfunction in insulin’s activation of the PI3K-Akt pathway. This correlation has been substantiated in both animal models and clinical observations of patients with T2DM (42, 43). In scenarios of IR, obesity, and T2DM, pinpointing the most impactful factor on cardiac health is challenging due to the associated metabolic disturbances. Nonetheless, evidence from mouse models devoid of myocardial insulin receptors strongly indicates a direct association between impaired glucose uptake, mitochondrial dysfunction, and contractile issues with disruptions in myocardial insulin signaling (19).

It is noteworthy that these mice also exhibited elevated mitochondrial ROS levels, potentially related to the inhibition of mitochondrial ETC proteins (44). Furthermore, some studies suggest that superimposed diabetes may enhance the production of mitochondrial ROS, possibly through an increased capacity to induce FAO, thus heightening the electronic load on the ETC, leading to electronic leakage and an upsurge in ROS production (45). Therefore, the adaptive regulation of compromised insulin signaling by myocardial metabolism may result in increased mitochondrial ROS levels in the heart, further exacerbating myocardial damage.

#### 3.2.3 Increased FA oxidation

In diabetic hearts, reduced glucose utilization by myocardial cells due to impaired insulin signaling or diminished glucose uptake (46) shifts the primary energy substrate to FAs, leading to an increase in FAO. This metabolic shift causes an excessive accumulation of acyl CoA, which not only boosts ROS production in both the cytoplasm and mitochondria but may also trigger various metabolic disorders (20). Prolonged exposure to the FA palmitate has been demonstrated to promote ROS generation and increase mitochondrial fission, further exacerbating mitochondrial dysfunction (47). Notably, accumulated acyl CoA in diabetic hearts can be repurposed for triglyceride synthesis or transformed into ceramide and other derivatives. Ceramide, known to directly inhibit ETC complex III, increases ROS production and may also initiate inflammatory responses, thus aggravating the progression of diabetic heart disease (19).

### 3.3 Effects of ROS in the myocardium

Regardless of source, elevated ROS levels damage nucleic acids, proteins, and lipids, causing cellular dysfunction and potentially cell death in diabetic cardiomyocytes.

#### 3.3.1 Lipid peroxidation

Unsaturated FAs are vulnerable targets for ROS, and lipid peroxidation (LPO) is a primary damage process induced by ROS. Malondialdehyde (MDA), a lipid peroxidation product, is an important biomarker of OS. Studies have indicated that levels of lipid peroxides in the hearts and serum of diabetic patients and animal models are significantly increased (48, 49). Furthermore, hyperglycemia and hyperlipidemia exacerbate lipid peroxidation reactions in the heart or myocardial cells (19, 50). It is noteworthy that enzymes inhibiting ROS generation (39), activating antioxidant systems, or enhancing ROS clearance mechanisms can effectively reduce MDA levels (51), thereby mitigating the effects of lipid peroxidation. Specifically, increased myocardial lipid peroxidation may be closely associated with lipid overload. Under sustained lipid peroxidation, proteins also undergo oxidative modification (52), particularly the FAD-containing subunit of succinate dehydrogenase, which may lead to mitochondrial dysfunction and further result in myocardial contractile damage in diabetes (53).

#### 3.3.2 Intracellular calcium balance

Unbalanced Ca²⁺ regulation is a significant characteristic of DCM. Although the mechanisms involved remain unclear, ROS oxidatively modify calcium ion channels on the myocardial cell membrane, including L-type calcium channels (LTCC) and calcium pumps, altering their function and disrupting calcium entry and release dynamics (54). ROS also activates calcium release channels, such as RyR2 in the SR, through oxidative reactions, promoting calcium release and thereby impairing the myocardium’s contractile and diastolic functions (54, 55). Furthermore, myocardial cells maintain calcium ion balance through calcium pumps, such as SERCA2a, and calcium-sodium exchange proteins (NCX). ROS oxidation of these proteins may decrease the activity of calcium pumps or alter the direction of calcium-sodium exchange, leading to obstructed calcium removal, potentially causing calcium overload or loss, and affecting myocardial contractility (54).

#### 3.3.3 Changes in mitochondrial membrane potential

Under normal conditions, the mitochondrial membrane potential maintains a high electrochemical gradient essential for ATP synthesis (56) and plays a critical role in intracellular calcium ion homeostasis (54). Excessive ROS damages mitochondrial proteins and lipids through oxidation, disrupting mitochondrial membrane integrity and leading to loss of membrane potential. This impairment directly affects ATP synthesis, resulting in inadequate cellular energy supply and potentially triggering cellular apoptosis or necrosis (20). Changes in membrane potential also influence calcium ion balance within mitochondria, promoting intracellular calcium ion accumulation, increasing OS responses, forming a vicious cycle, and exacerbating myocardial function decline (54). Thus, changes in mitochondrial membrane potential are central to ROS-induced myocardial dysfunction.

## 4 The interaction between mitochondrial OS and MF

### 4.1 Mitochondrial OS promotes MF

#### 4.1.1 Mitochondrial OS activates multiple signaling pathways

**Figure 2:**
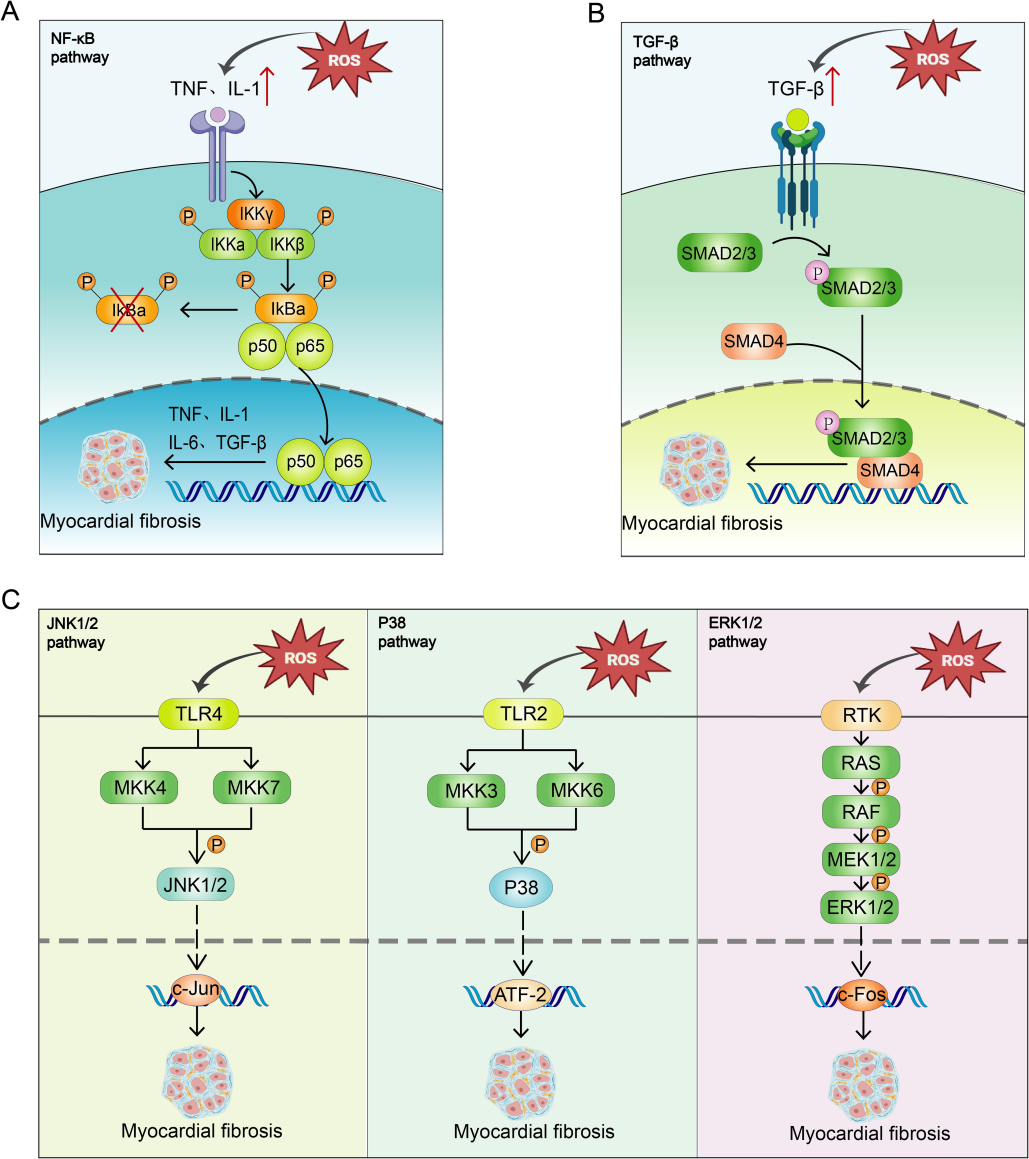
Mitochondrial OS Activates Multiple Pathways. **(A)** OS activates the NF-κB pathway, promoting the expression of inflammatory factors such as TNF-α and IL-1β, activating the IκB kinase complex, phosphorylating IκBα, and facilitating the entry of NF-κB (p50-p65) into the nucleus to regulate gene transcription, thus promoting MF. **(B)** OS activates the TGF-β/Smad pathway by promoting phosphorylation of Smad2/3, which forms a complex with Smad4 and translocates into the nucleus, regulating transcription of fibrosis-related genes and promoting MF. **(C)** OS activates MKK4/MKK7 via TLR4 receptors, thereby activating the JNK pathway. JNK enters the nucleus to regulate transcription factors such as c-Jun, promoting fibroblast activation and collagen synthesis. OS also activates MKK3/MKK6 via TLR2 receptors and activates the p38 MAPK pathway. P38 phosphorylation regulates ATF2 transcription factor, activating fibroblasts and promoting collagen synthesis; OS activates the ERK pathway, which promotes fibroblast proliferation and migration by phosphorylating c-Fos. These cells migrate to the damaged area to synthesize collagen and ECM, promoting fibrosis.

Mitochondrial OS impairs myocardial cell function by activating apoptosis or fibrosis-related pathways. ROS generated by mitochondrial OS react with NO to form hydroxyl radicals, damaging proteins, lipids, and DNA, altering the overall gene expression program of diabetic cardiomyocytes, and reducing myocardial contractility (57). Mitochondrial OS has been shown to activate multiple pathways (**Figure 2**).

Excessive ROS production occurs due to hyperglycemia, hyperlipidemia, and, compounded by inadequate endogenous antioxidant defense mechanisms. This imbalance results in mitochondrial dysfunction and persistent ROS accumulation. Consequently, key inflammatory pathways, such as NF-κB and the NLRP3 inflammasome, are activated, prompting immune cells such as macrophages and neutrophils to secrete inflammatory cytokines (IL-1β, TNF-α, IL-6, TGF-β1), thus perpetuating chronic inflammation (9, 58).

##### 4.1.1.1 NF-κB pathway

The NF-κB pathway plays a pivotal role in OS-induced MF. In diabetic conditions, excessive ROS activate the NF-κB pathway, promoting the expression of pro-inflammatory factors such as TNF-α and IL-1. These cytokines, through their respective receptors TNFR and IL-1R, activate the IκB kinase (IKK) complex, which consists of IKKα, IKKβ, and IKKγ. Upon activation, this complex phosphorylates IκB proteins, primarily IκBα in the cytoplasm, which normally prevent the NF-κB dimer (p50-p65) from entering the nucleus. Phosphorylated IκB is subsequently degraded via the ubiquitin-proteasome system, allowing the NF-κB dimer to migrate to the nucleus and bind to DNA, influencing protein transcription, damaging myocardial cells, and promoting fibroblast activation and migration (54, 59). Furthermore, the NF-κB pathway supports the transformation of fibroblasts into myofibroblasts by regulating transcription factors such as TGF-β and Smad, increasing collagen synthesis and ECM deposition (60), thereby enhancing the fibrosis process (54, 61).

##### 4.1.1.2 TGF-β/Smad pathway

TGF-β is integral to cardiac repair and remodeling, influencing the phenotype and functionality of cardiomyocytes, fibroblasts, immune cells, and vascular cells (62). Activated in both experimental models of cardiac fibrosis and human fibrotic hearts (63), an increase in ROS levels induced by OS enhances the synthesis and secretion of TGF-β. The Smad protein, acting as a downstream effector, is involved in this pathway (64). TGF-β, upon binding to its receptors on the cell surface, activates kinase activity that phosphorylates Smad proteins. Phosphorylated Smad2/3 forms a complex with Smad4 and translocates into the nucleus to regulate the transcription of fibrosis-related genes such as collagen, fibronectin, and integrins (64). This TGF-β/Smad pathway substantially contributes to MF, promoting the transformation of fibroblasts into myofibroblasts (65, 66), enhancing their collagen and ECM synthesis capabilities, leading to ECM accumulation, which stiffens cardiac tissue and impairs cardiac function (66). Additionally, this pathway enhances myofibroblast contractility, further advancing MF (66).

##### 4.1.1.3 MAPK pathway

The Mitogen-activated protein kinase (MAPK) pathway, a highly conserved serine/threonine protein kinase cascade (67). encompasses four branches: ERK, ERK5, JNK, and p38/MAPK. Activated by OS, these pathways govern cellular processes such as proliferation, migration, apoptosis, inflammatory responses, and collagen synthesis, contributing significantly to MF (68). These pathways individually stimulate fibroblast activation, collagen synthesis, inflammatory responses, and apoptosis through distinct mechanisms, driving the progression of MF.

###### 4.1.1.3.1 JNK pathway

In cardiac fibroblasts, extracellular stimuli such as OS activate the MKK4/MKK7 kinase via the TLR4 receptor on the cell membrane, which in turn activates the JNK pathway. Upon activation, JNK enters the nucleus to regulate transcription factors like c-Jun, inducing the synthesis of pro-inflammatory factors that activate fibroblasts and promote collagen synthesis (67).

##### 4.1.1.3.2 p38 MAPK pathway

Extracellular stimuli such as OS activate MKK3/MKK6 kinases via the TLR2 receptor, subsequently activating the p38 pathway (69). Phosphorylated p38 MAPK enters the nucleus to regulate ATF2, activating fibroblasts and promoting collagen synthesis (70, 71).

##### 4.1.1.3.3 ERK pathway

ERK1 and ERK2 share 83% similarity and most signaling functions, commonly referred to as ERK1/2. OS increases the secretion of growth factors, which bind to receptors and activate receptor tyrosine kinase (RTK) on the cell membrane. This activation triggers Ras and further activates MEK (MAPK/ERK kinase) (72), ultimately activating the ERK pathway. The ERK signaling pathway phosphorylates the downstream transcription factor c-Fos (73), promoting fibroblast proliferation and migration. Fibroblasts migrate to the damaged area and synthesize collagen and other ECM components, leading to fibrosis (67). Additionally, the ERK pathway facilitates the transformation of fibroblasts into myofibroblasts (67), which are crucial in the fibrosis process and capable of synthesizing significant amounts of collagen, particularly type I and III collagen, exacerbating the fibrosis process.

#### 4.1.2 Mitochondrial OS induces uncoupling

Accumulated mitochondrial ROS activate uncoupling proteins (UCP) in the mitochondrial inner membrane (74), promoting proton reflux from the mitochondrial intermembrane space to the matrix, diminishing the proton gradient required for OXPHOS, reducing ATP synthesis, and releasing metabolic energy as heat (75). Mild mitochondrial uncoupling mediated by UCPs moderately reduces mitochondrial membrane potential, thus lowering electron leakage at complexes I and III and subsequently reducing ROS generation, exerting antioxidant effects on myocardial cells and protecting them from oxidative damage (74). However, under pathological conditions such as HF or diabetes, excessive uncoupling leads to ATP synthesis reduction, resulting in energy loss primarily as heat, causing insufficient energy supply to myocardial cells, metabolic disorder, and mitochondrial dysfunction. Continuous ROS accumulation not only aggravates OS but also affects myocardial contraction and relaxation, escalating cardiac strain and potentially promoting persistent MF (76). Modulating the expression of uncoupling proteins like UCP2 and UCP3 can mitigate MF occurrence while preserving cellular antioxidant capabilities (77).

#### 4.1.3 Mitochondrial OS damages mitochondrial DNA

Mitochondrial OS results in an excessive generation of ROS, inflicting oxidative harm to mitochondrial DNA (mtDNA) and its encoded proteins, causing cellular dysfunction and inner mitochondrial membrane damage (20). This damage reduces the mitochondrial membrane potential, decreases ATP synthesis capability, and increases ROS production, creating a vicious cycle that further exacerbates mtDNA damage (20). Mitochondrial DNA, lacking histone protection and possessing weaker DNA repair mechanisms compared to nuclear DNA, is more vulnerable to OS. Damage to mtDNA severely affects the function of the mitochondrial respiratory chain, which relies heavily on mtDNA-encoded proteins. Once mtDNA repair is compromised, damaged DNA cannot be effectively repaired (78). Damaged mtDNA activates apoptotic signaling pathways in cells. Dysfunctional mitochondria and damaged mtDNA release cytokines into the cytoplasm, inducing caspase-dependent apoptosis, and leading to cardiomyocyte apoptosis. Apoptotic cardiomyocytes release damaging factors, exacerbating damage to surrounding tissues and promoting pathological changes in the heart (79). Long-term mtDNA damage not only leads to mitochondrial dysfunction but also has irreversible effects on the physiological functions of myocardial cells, ultimately inducing a series of CADs, especially MF.

#### 4.1.4 Mitochondrial OS disrupts mitochondrial membrane permeability

Moreover, mitochondrial OS induces oxidative damage to mitochondrial membranes and lipids, compromising the structure of the mitochondrial inner membrane, thereby diminishing its fluidity and stability and affecting its permeability. This damage not only leads to endometrial dysfunction but also alters membrane permeability, impacting cellular function (80, 81). Crucially, ROS regulate mitochondrial permeability transition pore (mPTP) opening. Excessive ROS production, triggered by hyperglycemia, prolongs mPTP opening duration (82), causing mitochondrial membrane potential depolarization, reversal of ATP synthase transport, and release of pro-apoptotic factors such as cytochrome c and TNF, thus initiating caspase cascades. Caspases, involved in cell growth, differentiation, and apoptosis regulation, accelerate myocardial apoptosis (83). Additionally, mPTP opening allows water into mitochondria, leading to swelling or rupture, reducing energy supply, and further promoting cardiomyocyte apoptosis (84). The buildup of mitochondrial ROS also heightens mitochondrial membrane permeability, mediates lipid peroxidation and protein carbonylation, intensifies OS, creates a destructive cycle, and ultimately leads to cell apoptosis or necrosis, thus fostering MF (85).

#### 4.1.5 Mitochondrial OS affects mitochondrial autophagy

Mitochondrial OS reduces mitochondrial autophagy levels, promoting cell apoptosis and exacerbating MI. Mitochondrial autophagy is crucial for repairing mitochondrial function and maintaining cellular homeostasis by transporting damaged or dysfunctional mitochondria to lysosomes for degradation (86). Typically activated by nutrient deficiency or decreased mitochondrial membrane potential, mitophagy involves PINK1 and its substrate Parkin as key components (20). Effective in removing damaged or aging mitochondria, mitochondrial autophagy blocks harmful mitochondrial signals and recovers small molecules such as glucose, amino acids, nucleotides, phospholipids, and FAs, maintaining cellular energy balance (20). In diabetes patients and their animal models, significant changes in the expression of mitochondrial autophagy-related genes and proteins have been observed. In the hearts of diabetic mice, mitochondrial autophagy activity is notably reduced (87) and the PINK1/Parkin pathway is inhibited (88). High glucose conditions further reduce mitochondrial autophagy levels due to ROS accumulation, leading to increased apoptosis, inhibited cell proliferation, and downregulation of autophagy-related proteins such as PINK1 and Parkin. The downregulation can be reversed by ROS scavengers such as N-acetyl-L-cysteine (NAC) (89), highlighting ROS’s critical role in impairing mitophagy. Impaired mitophagy contributes to mitochondrial dysfunction, lipid accumulation, and promotes MF. Conversely, activating mitophagy alleviates MF, underscoring its potential therapeutic value in preventing and treating CADs.

#### 4.1.6 Mitochondrial OS accumulates metabolites

In diabetes patients, substantial amounts of metabolic substances such as acyl CoA and acylcarnitine affects mitochondrial ATP/ADP ratios, impairing metabolic function. Elevated glucose promotes lipid and protein oxidation through mitochondrial OS, increasing advanced glycation end products (AGEs), thereby altering myocardial structure (37). AGEs disrupt insulin metabolism signaling, diminish NO production, and augment cardiac fibrosis. They bind to the receptor for glycation end products (RAGE), activate the MAPK pathway, initiate inflammatory responses, promote matrix protein synthesis, and connective tissue growth, leading to MI (37). Further studies indicate that increased ROS in diabetic hearts activate peroxisome proliferator-activated receptor alpha (PPARα), enhance FA uptake and oxidation, result in the accumulation of metabolic by-products such as AGEs, provoke inflammatory responses, and ultimately cause MF (37).

### 4.2 MF promotes mitochondrial OS

#### 4.2.1 Accumulation of ECM increases OS

During MF, fibroblasts secrete large quantities of collagen and other ECM components, forming fibrotic tissue. ECM deposition and cardiac fibrosis increase the distance between capillaries and myocardial cells, leading to reduced oxygen diffusion efficiency. Excessive ECM accumulation also causes hardening and structural changes in myocardial tissue, impairing normal blood circulation, especially in microvessels, and putting the myocardium at risk of hypoxia (90, 91). In heart tissues of diabetic animals and humans, the expression of VEGF and its receptors is downregulated, exacerbating hypoxia and leading to severe damage (90). This results in reduced ATP production, impaired mitochondrial function, and activation of ROS generation pathways, including NADPH oxidase. Increased ROS levels further damage myocardial cells and promote fibroblast activation, forming a vicious cycle.

#### 4.2.2 Activation of fibroblasts increases OS

During MF, fibroblasts are stimulated by various growth factors such as TGF-β, Ang II, PDGF, and transform into myofibroblasts. Myofibroblasts, with their enhanced ability to synthesize ECM, secrete more ROS. These ROS not only damage myocardial cells but may also promote further fibroblast proliferation and activation through paracrine effects, thus forming a vicious cycle (92).

#### 4.2.3 Inflammatory response and signaling pathways increase OS

Fibrosis often coincides with an inflammatory response, with TGF-β acting as a primary regulator in both *in vivo* and *in vitro* fibrosis scenarios, closely linked to MF (93). Macrophages, significant sources of TGF-β1 when activated by interferon-κ (IFN-κ), exhibit enhanced NF-κB activity. These macrophages also manifest pro-inflammatory phenotypes, generating a variety of chemokines and ROS, which precipitate a vicious cycle of tissue damage and fibrosis (94). Macrophage depletion diminishes fibrosis post-injury, whereas their recruitment increases fibrotic lesions. Hence, the infiltration and activation of inflammatory cells likely drive the initiation and progression of fibrotic diseases. Mounting evidence suggests inflammation is intricately connected to fibrosis, playing a substantial role in the pathogenesis of diabetes and heart disease (95). Characteristics of fibrosis include heightened inflammatory response, tissue degradation, and activation of signaling pathways such as NF-κB and JNK, which facilitate the infiltration of inflammatory cells and the release of mediators including TGF-β1, tumor necrosis factor-alpha (TNF-α), monocyte chemoattractant protein-1 (MCP-1), interleukin-6 (IL-6), and IL-8. These factors not only exacerbate OS but also further activate fibroblasts, promoting ECM synthesis and deposition, and accelerate fibrosis progression (92).

#### 4.2.4 Mitochondrial damage increases OS

Mitochondrial damage is common during MF, significantly impacted by OS, which reduces mitochondrial membrane potential and activate mPTP, exacerbating intracellular calcium ion accumulation and further increasing OS (83). Mitochondrial damage also leads to ROS accumulation within cells, further harming cardiac myocytes. During MF, mitochondrial autophagy may be impaired due to ROS accumulation and cytokine involvement, hindering the timely clearance of damaged mitochondria, leading to increased ROS generation and heightened OS (89).

#### 4.2.5 Cellular metabolic changes enhance OS

During MF, ECM deposition and cardiac fibrosis worsen the heart’s hypoxic conditions. Under insufficient oxygen supply, mitochondria resort to anaerobic metabolic pathways, such as anaerobic glycolysis, to produce energy, which is significantly less efficient than aerobic metabolism. Excessive accumulation of lactic acid as a metabolic byproduct may be toxic to cells, impairing cellular function. In this metabolic state, more ROS are produced. Hypoxia and an increase in ROS not only damage mitochondria but also lead to the activation of more fibroblasts and the synthesis of ECM, forming a vicious cycle (96).

### 4.3 Mitochondrial OS and MF interact with each other, forming a vicious cycle

**Figure 3:**
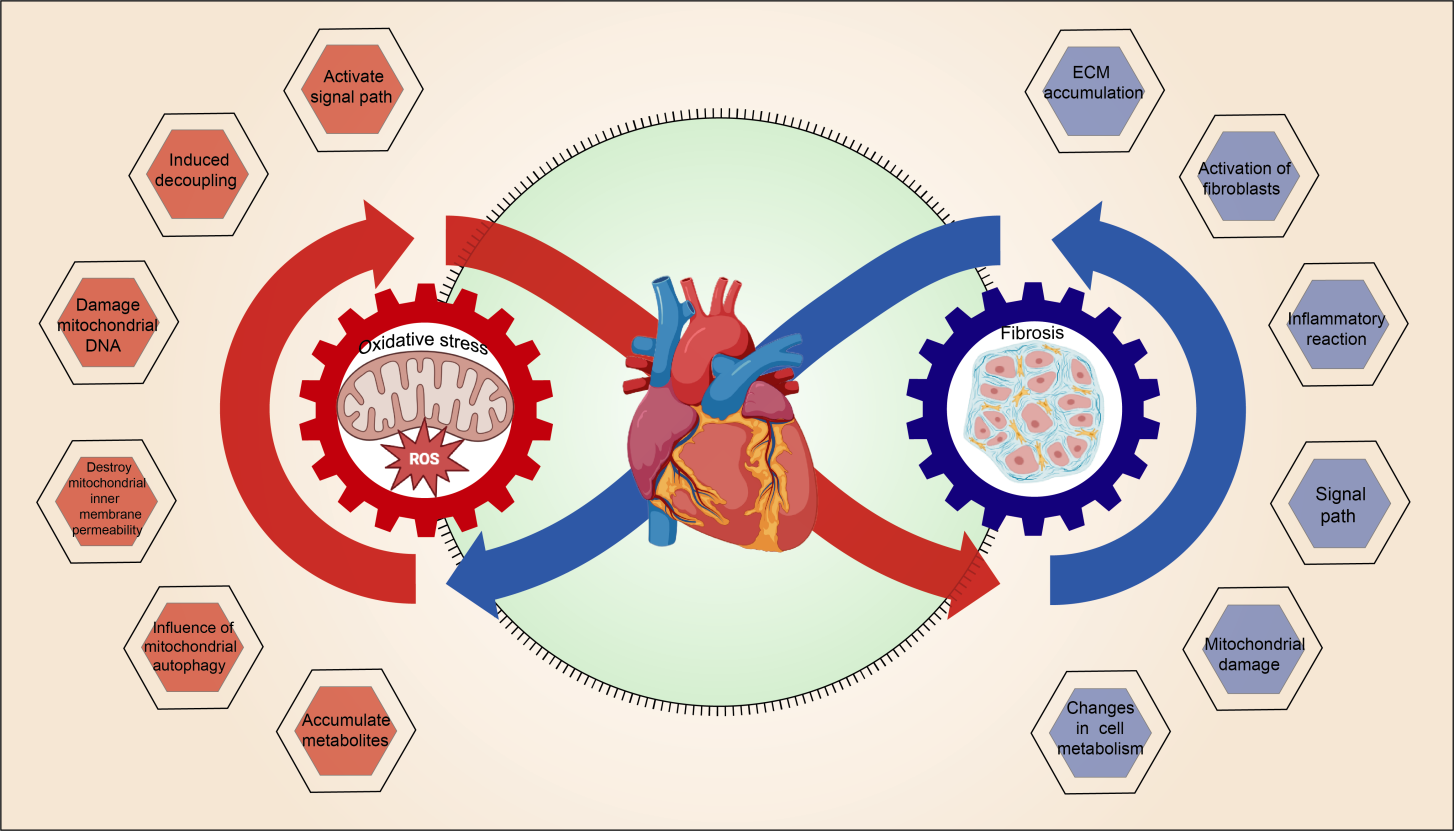
The vicious cycle of mitochondrial OS and MF.

The interaction between mitochondrial oxidative stress and myocardial fibrosis, which forms a vicious cycle, is illustrated in **Figure 3**.

### 4.4 Summarize current clinical and animal model research

Mitochondrial OS represents a pivotal mechanism driving MF associated with diabetes. Elevated ROS generation through OS can stimulate fibroblast activation, enhance extracellular matrix (ECM) deposition, exacerbate inflammation, and damage mitochondria, ultimately promoting MF. As summarized in **Table 1**, OS has been established as an essential contributor to diabetic MF in both animal models and clinical studies.

**Table 1:** Summarize existing animal experiments and clinical research data to elucidate the relationship between OS and fibrosis.

Animal Experiments and Clinical Research			
Serial Number	Animals/Humans	Research Results	Quote
1	C57BL/6 mice and db+/- mice	Therapeutic inhibition of mito TEMPO on mitochondrial ROS reduces MF in diabetes mice	Ni, Rui et al (97)
2	Male C57BL/6 mice	Enhanced MAO-A activity leads to increased generation of ROS, resulting in MF and left ventricular dysfunction	Kaludercic, Nina et al (98)
3	SD rats	MAO-A induced OS is the cause of 5-HT induced apoptosis and fibrosis in cardiomyocytes	Bianchi, Pascale et al (99)
4	*C-Nox4-/- Mouse*	Nox4 in cardiomyocytes is the main source of mitochondrial OS, and its overexpression causes myocardial dysfunction, fibrosis, and cell apoptosis.	Kuroda, Junya et al (100)
5	*Sod2 (-/+) mice*	Knockout of MnSOD promotes MPTP activation and ROS induced myocardial cell fibrosis and apoptosis in cardiac mitochondria	Van Remmen, H et al (101)
6	*Sod2 (-/+) mice*	MnSOD homozygous knockout leads to left ventricular dilation, myocardial cell hypertrophy, and fibrosis	Li, Y et al (102)
7	OVE26 diabetes mice	Overexpression of MnSOD reduces OS and inhibits MF	Shen, Xia et al (103)
8	Male Gpx1-/- mice	Gpx1 loss increases OS and promotes MF	Ardanaz, Noelia et al (104)
9	GSHPx transgenic (TG) mice	Overexpression of Gpx1 reduces OS and inhibits MF	Shiomi, Tetsuya et al (105)
10	Male Prx-3 transgenic mice	Overexpression of Prx3 interferes with mitochondrial OS, reduces myocardial hypertrophy and fibrosis	Matsushima, Shouji et al (106)
11	MCAT overexpressing mice	Mitochondrial targeted antioxidants can alleviate OS and reduce MF	Dai, Dao-Fu et al (107)
12	WT and KO mice	The deletion of p66shc gene reduces OS and improves MF	Rota, Marcello et al (108)
13	C57BL/6J mice	Exendin-4 delays MI and fibrosis in diabetes mice by improving mitochondrial function and inhibiting OS	Cai, Ying-Ying et al (109)
14	olgA overexpressing mice	Mitochondrial DNA point mutations and deletions promote MF	Trifunovic, Aleksandra et al (110)
15	STZ induced diabetes mice	Mitochondrial dysfunction promotes MF	Becher, P M et al. (111)
16	Zucker diabetes rats	Mitochondrial dysfunction promotes MF	Huang, Tom H W et al. (112)
17	human	Engrelizin reduces OS, inhibits inflammation, and fibrosis by differentially expressing circulating proteins in HF	Zannad, Faiez et al (113)
18	human	Mitiberstat reduces OS, decreases MF and remodeling. Improve symptoms and prognosis of HF	Lund, Lars H et al (114)
19	human	Quentin reduces OS, lowers LVH, and fibrosis	Farrant, John et al (115)
20	human	NAC is an antioxidant with a relatively small impact on cardiac fibrosis, possibly due to the small sample size that hinders definitive conclusions	Marian, Ali J et al (116)
21	human	SVAP-1 promotes liver fibrosis by catalyzing the generation of ROS	Weston, Chris J et al (117)
22	human	Antioxidant N-acetylcysteine reduces OS and delays the progression of pulmonary fibrosis	Tomioka, Hiromi et al (118)
23	human	β - carotene reduces OS, decreases pulmonary fibrosis, and improves lung function	Wood, Lisa G et al (119)
24	human	Glutathione reduces OS and has a slight positive effect on lung function, which deserves further research	Calabrese, C et al (120)
25	human	Selenium and coenzyme Q10 have synergistic antioxidant properties, reducing fibrosis and improving myocardial function	Alehagen, Urban et al (121)
26	human	Losartan blocks renal OS and pro-inflammatory state in CKD patients, reducing renal fibrosis	Agarwal, Rajiv et al (122)
27	human	Autopsy shows an increase in type III collagen content	Shimizu, M et al (123)

However, there are inherent limitations in animal and clinical research. Although animal models are indispensable for elucidating fundamental biological mechanisms, they cannot fully replicate human disease complexity, particularly regarding drug metabolism, immune responses, and physiological traits. In human research, factors such as genetic background, lifestyle diversity, environmental influences, and individual variations limit the generalizability and consistency of findings. Moreover, clinical trials often face challenges related to sample size and heterogeneity, potentially compromising result reliability. Nevertheless, ongoing advancements in scientific technology and interdisciplinary collaboration will gradually overcome these hurdles, thereby improving precision medicine strategies for human health. 

## 5 Treatment strategies

Lifestyle modification is a critical non-pharmacological approach for managing diabetes. Stringent glucose control can mitigate the adverse effects of hyperglycemia on the heart, and increased physical activity—through aerobic exercises such as walking, swimming, and cycling—helps burn calories, enhances cardiopulmonary function, improves insulin sensitivity, and aids blood glucose management (20). Treatment also includes antioxidant therapy and targeted mitochondrial therapy (**Table 2**).

**Table 2:** Summarizes the mechanisms, efficacy, and clinical research progress of antioxidant therapy and targeted mitochondrial therapy in current literature.

Treatment				
	Medicant	Mechanism	Effect on the cardiac	Quote
Antioxidant therapy				
1	vitamin C	Water soluble free radical scavenging antioxidant	Invalid or harmful	Peoples, Jessica N et al (126)
2	vitamin E	Stable lipid peroxidation free radicals	Invalid	Peoples, Jessica N et al (126)
3	SOD	Antioxidant enzymes that clear ROS	Reduce oxidative damage and cardiac fibrosis	Khattab, Elina et al (125)
4	NAC	Thiol containing antioxidants	Reduce myocardial damage caused by OS, improve the survival rate and function of myocardial cells	Peoples, Jessica N et al (126)
5	Nrf2 activator	Enhance antioxidant defense	Reduce oxidative damage and cardiac fibrosis	Huynh, Karina et al (131)
Targeted mitochondrial therapy				
1	Sirtuin 3	Enhance mitochondrial function and reduce OS	Reduce OS damage and mitochondrial dysfunction	Kandy, Amarjith Thiyyar et al (132)
2	coenzyme Q10	Enhance mitochondrial antioxidant capacity	Reduce left ventricular size and collagen deposition	Arad, Michael et al (127)
3	Peroxyreductase protein-3 (Prx-3)	Enhance mitochondrial antioxidant capacity	Reduce myocardial cell damage induced by hyperglycemia	Arad, Michael et al (127)
4	Mitochondria TEMPO	Enhance the clearance of mitochondrial ROS	Reduce myocardial cell damage induced by hyperglycemia	Peoples, Jessica N et al (126)
5	MitoQ	Reduce OS and improve mitochondrial function	Reduce myocardial hypertrophy and fibrosis	Peoples, Jessica N et al (126)
6	SS-31	Reduce OS and improve mitochondrial function	Inhibit myocardial apoptosis and fibrosis	Peoples, Jessica N et al (126)
7	EGCG	Reduce OS and inflammation	Regulating autophagy pathway to protect myocardial cells	Khattab, Elina et al (125)
8	mitochondrial transplantation	Transplanting healthy mitochondria into damaged tissues or cells	Reduce OS and improve mitochondrial function	Hassanpour, Parisa et al (133)

### 5.1 Antioxidant therapy

NADPH oxidase significantly contributes to myocardial cell hypertrophy, fibrosis, and the activation of pro-fibrotic pathways (54, 124). Diabetes patients often experience increased OS, leading to an accumulation of free radicals that worsen MI. OS escalates ROS production, activates fibroblasts, promotes ECM deposition, intensifies inflammatory responses, damages mitochondria, and ultimately leads to MF. Thus, antioxidant therapy is viewed as a potentially effective strategy to alleviate MF in diabetes. Common antioxidant treatments encompass SOD activators, NAC, and Nrf2 activators such as resveratrol, which mitigate oxidative damage, enhance mitochondrial function, and prevent cardiac fibrosis and hypertrophy (125). The effectiveness of vitamins E and C in clinical trials remains debated, with most studies indicating limited benefits regarding cardiovascular event reduction (126).

### 5.2 Targeted mitochondrial therapy

Considering the limited efficacy of general antioxidants in addressing heart disease and the significant potential of targeted mitochondrial therapy, extensive research has focused on this area. This includes sirtuin 3 activators, Prx-3, coenzyme Q10, and mitochondrial-targeted antioxidants (e.g., mitoTEMPO), all of which bolster antioxidant defenses to counter mitochondrial OS (127). Coenzyme Q10 has been shown to reduce left ventricular (LV) size and collagen deposition, while mito-TTEMPO and Prx-3 protect cardiomyocytes against high glucose-induced damage (125, 128). MitoQ reduces myocardial oxidative damage and improves mitochondrial membrane integrity (129). SS-31 enhances mitochondrial function, reduces intracellular OS, decreases cardiomyocyte apoptosis, and inhibits MF, thereby protecting the myocardium (130). Additionally, epigallocatechin gallate (EGCG) promotes mitochondrial autophagy, clears damaged mitochondria, reduces cellular stress, and enhances cellular metabolic function (125).

### 5.3 Summarize current treatment plans

## 6 Discussion, conclusion, future prospects, and challenges

This review underscores that the interplay between mitochondrial OS and MF is increasingly recognized as pivotal in cardiovascular research within the diabetes context. Patients with diabetes frequently suffer from mitochondrial dysfunction and heightened OS response, substantially contributing to myocardial cell damage and the progression of MF. Mitochondrial OS activates various signaling pathways, including NF-κB, TGF-β/Smad, and MAPK pathways. Complex cross-regulatory interactions, such as positive feedback between NF - κ B and TGF - β signaling and MAPK-mediated phosphorylation of Smad proteins, along with epigenetic and post-translational modifications, disrupt ECM homeostasis. These processes establish a detrimental feedback cycle of OS, chronic inflammation, and fibrotic remodeling, promoting fibroblast proliferation, collagen deposition, ECM accumulation, and ultimately MF and cardiac dysfunction. A deeper comprehension of the deleterious cycle between OS and MF can elucidate the mechanisms through which diabetes induces MF and offer a theoretical foundation for developing novel diagnostic and therapeutic approaches.

Future research should focus on elucidating how mitochondrial OS specifically contributes to MF in diabetes through molecular mechanisms. It is essential to systematically unveil the network mechanisms of interactions between OS and other pathological factors such as inflammation, endothelial dysfunction, and metabolic disorders. Moreover, considering the individual differences among diabetes patients, identifying early markers of MF and integrating genomics, metabolomics, and other multi-omics data to advance research on early diagnosis and personalized treatment are crucial future directions.

Although several studies have explored methods such as antioxidants and mitochondria-targeted therapies to mitigate OS-mediated MI, their therapeutic effectiveness remains limited. Future therapeutic efforts should prioritize novel strategies that precisely regulate mitochondrial function with minimal adverse effects. Although emerging technologies such as gene editing, stem cell therapies, and mitochondrial transplantation offer promising therapeutic breakthroughs, their clinical translation is still hindered by practical challenges, such as ethical controversies, insufficient safety verification, and cost-effectiveness. Importantly, current research faces obstacles such as discrepancies between preclinical models and human pathology, experimental heterogeneity leading to insufficient data reproducibility, insufficient accuracy in drug screening, and inter-patient variability, collectively impeding the clinical translation of basic research findings. Therefore, optimizing the research paradigm through interdisciplinary collaboration is crucial: long-term follow-up data collection should be strengthened to dissect complex interactions between diabetes and MF, while integrating multi-omics analyses and artificial intelligence-assisted modeling could significantly enhance translational efficiency, ultimately providing innovative and feasible solutions for precision management of diabetic CVD. 
